# Ladd’s band in the adult, an unusual case of occlusion: Case report and review of the literature

**DOI:** 10.1016/j.ijscr.2020.04.046

**Published:** 2020-05-11

**Authors:** Carmine Grassi, Luigi Conti, Gerardo Palmieri, Filippo Banchini, Maria Diletta Dacco’, Gaetano Maria Cattaneo, Patrizio Capelli

**Affiliations:** a Department of Surgery, AUSL Piacenza, Via Taverna 49, 29121 Piacenza, Italy; b Department of Medicine and Surgery, AOU Parma, Via Gramsci 14, 43122 Parma, Italy

**Keywords:** Gut malrotation, Ladd’s band, Bowel obstruction

## Abstract

**Introduction:**

Malrotation of gut is a congenital anomaly of foetal intestinal rotation and it is mostly discovered in early childhood as acute intestinal obstruction. This condition is very rare and often silent in adults.

**Presentation of case:**

We present a case of 44-year-old woman admitted with acute abdominal pain. The abdominal CT scan showed ileal conglomerate loops with thickened walls filled of liquid in the right iliac side. Laparotomic Ladd's procedure was performed successfully with division of Ladd's band, adhesiolysis, reconstruction of Treitz's ligament, small bowel resection and Merckel's diverticulum removal. The patient made an unremarkable recovery.

**Discussion:**

We discuss the rarity of intestinal malrotation in adulthood and made a literature review comparison of the therapeutic options available.

**Conclusion:**

Intestinal malrotation is a rare entity and adult presentation is even rarer. Some cases are asymptomatic, but when symptomatic a volvulus should be promptly suspected to avoid complications such as bowel ischemia.

## Highlights

• Malrotation of gut is a congenital anomaly of foetal intestinal rotation and is very rare and often silent in adults.
• We performed a laparotomic Ladd’s procedure in a case of 44 year old woman with acute abdominal pain.
• Intestinal malrotation is a rare entity and adult presentation is even rarer.
• Some cases are asymptomatic, but when symptomatic a volvulus should be promptly suspected to avoid complications such as bowel ischemia.

## 1 Introduction

**Fig. 1:**
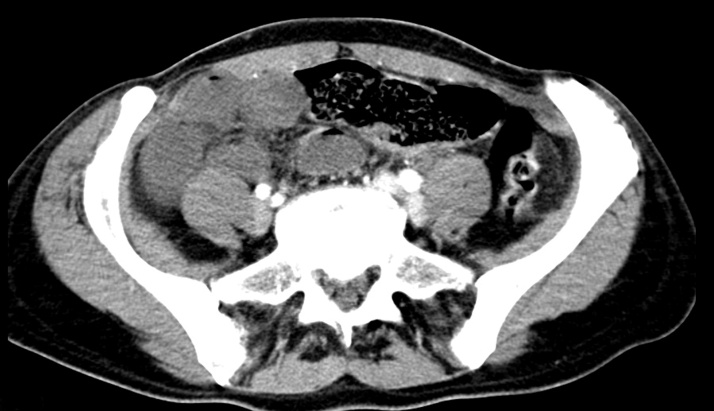
CT scan of the abdomen: conglutinated ileal loops in the right iliac fossa.

This work has been reported in line with the SCARE criteria [1]. Intestinal malrotation is caused by partial or complete failure of 270 degree counter clockwise rotation of midgut around superior mesenteric vessels in foetal life [2]. Malrotation in adult is very rare. Bilious vomiting, bowel obstruction, abdominal pain are classically described as presenting complains in intestinal malrotation [17]. Malrotation can be associated with other congenital anomalies in childhood. Ladd first described this procedure to treat malrotation and volvulus in 1932 and since then it has been the definitive treatment for intestinal malrotation [3,4]. Ladd’s procedure consists of initial untwisting of the volvulus. Secondly, Ladd’s bands (Fig. 1), thick peritoneal bands running from the caecum to the right upper quadrant and to the duodenum, are divided. The ligament of Treitz is taken down and the duodenum is mobilized to the right and straightened. The entire bowel is returned to the abdomen in a non-rotated position. In the majority of medical literature the focus is on correct and early identification of the symptoms of malrotation, appropriate investigation and a prompt Ladd’s procedure [5,6]. In neonates malrotation is a recognized risk factor for adhesions related obstruction [7]. Small bowel obstruction has been noted following Ladd’s procedure. A chronic malabsorbitive-like syndrome can occur in patients with chronic midgut volvulus [8].

## 2 Presentation of case

A 44 year old woman attended the emergency department, complaining acute abdominal pain in periumbilical, bilateral iliac and hypogastric regions. The routine blood tests showed: WBC 13120, Hemoglobin 11 g/dl, HCT 33,5, CRP within normal limit. She was admitted and managed with nasogastric tube decompression and intravenous fluid hydration for a suspected bowel obstruction. X ray abdomen showed signs of bowel obstruction with air-fluid levels of midgut; chest x-ray was unremarkable. The abdominal CT scan revealed an conglomerate ileal loops with thickened walls filled of fluid in the right iliac recess (Fig. 1).

**Fig. 2:**
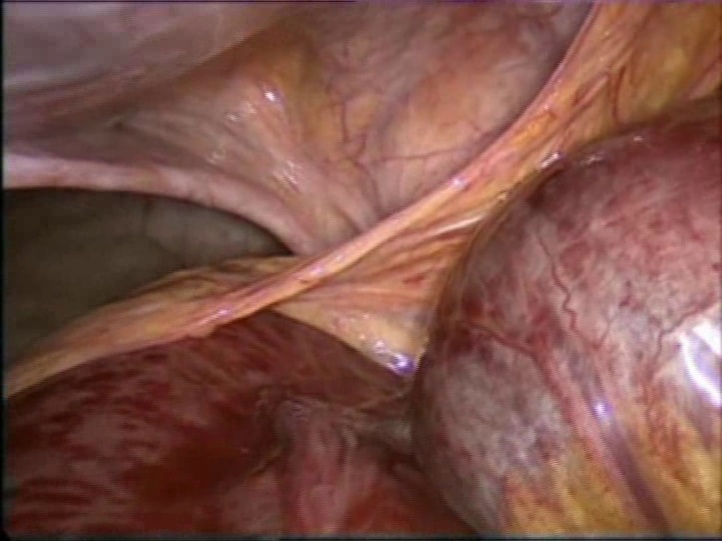
Endoscopic picture showing the fibrous band crossing the midline from the medial to right side anteriorly to ileum, with dilated incarcerated bowels.

**Fig. 3:**
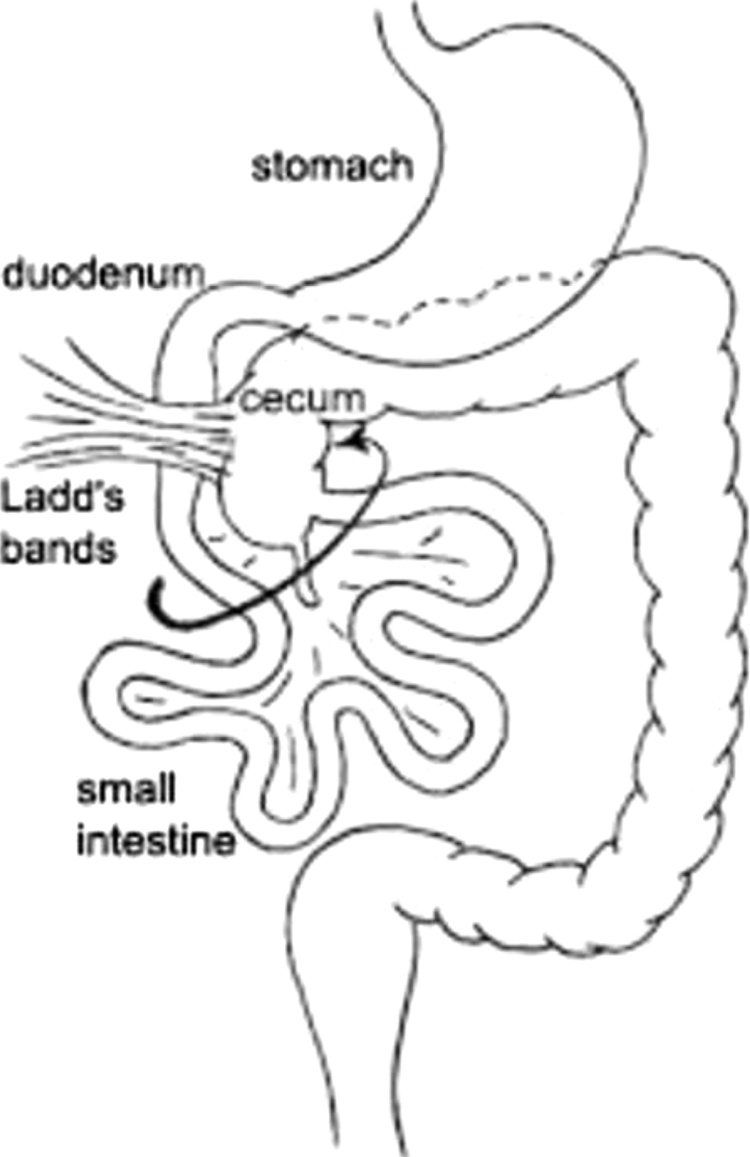
Depiction of the Ladd’s band caused by bowel malrotation.

**Fig. 4:**
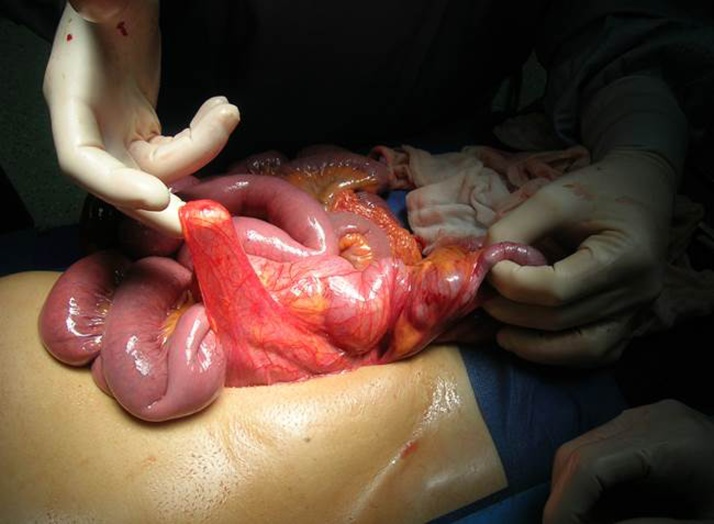
Intraoperative image representing the fibrous band during small bowel adhesiolisis.

A diagnosis of bowel obstruction was made and the patient sent for urgent laparoscopy. At laparoscopy (Fig. 2), the patient was found to have many visceral adhesions in right iliac fossa, small bowel distension, the complete absence of Treitz’s ligament and an intestinal volvulus with edematous, necrotic ileal loop in malrotation, showing the presence of a band between the right colon and the liver which divided the ileal loops located at right of ascending colon (Fig. 3, Fig. 4) so we decided to perform open surgery.

**Fig. 5:**
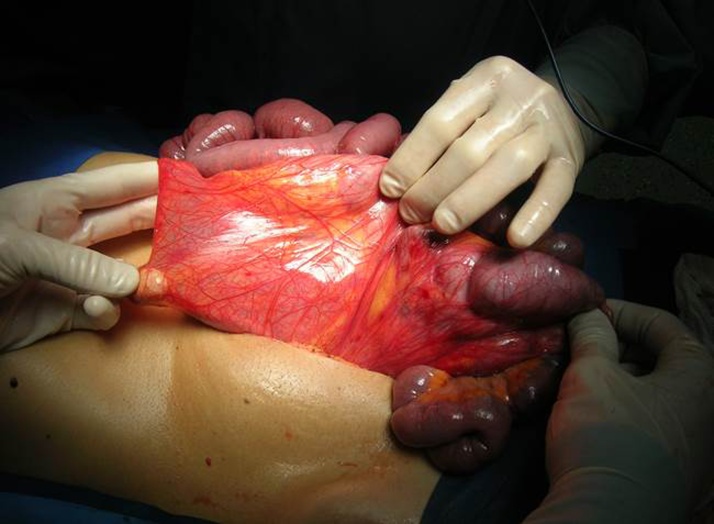
Intraoperative finding: Ladd’s band.

**Fig. 6:**
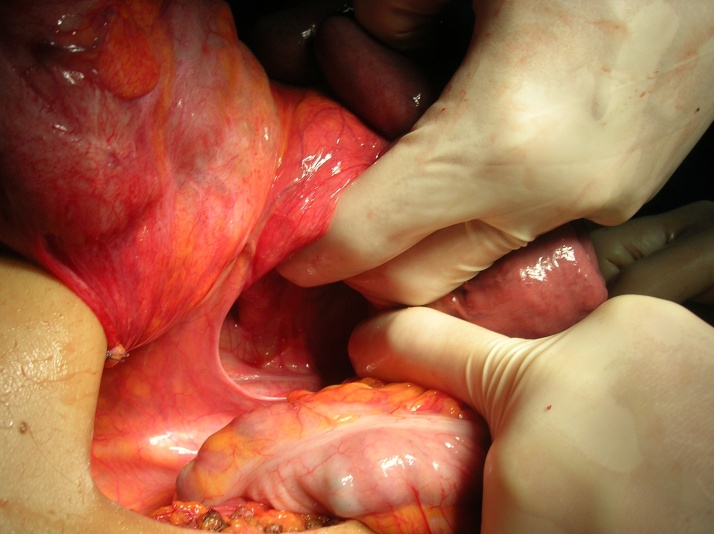
Intraoperative image: mesenteric orifice causing an internal hernia.

We first untwisted the volvulus, then returned the bowels in the abdomen in a non-rotated position. We performed the closure of the internal mesenteric passageway (Fig. 5, Fig. 6) then the resection of necrotic small bowel segment, the section of the fibrous band and the resection of Meckel diverticulum incidentally find on exploration. The patient made an unremarkable recovery and was discharged on fifth post-operative day. We didn’t reported any recurrence of symptoms after one year follow-up.

## 3 Discussion

The normal development of mid-gut occurs in three stages of rotation process between the 4th and 12th week of embryonic period. During this phase duodenum is fixed retro-peritoneally at the ligament of Treitz in the left and caecum on right lower abdomen. Malrotation of the gut is due to complete or partial failure of 270 degree of clockwise rotation of the midgut around the superior mesenteric pedicle; it results in anomalous position of small bowel loops in the right side with absence of ligament of Treitz and appendix, caecum and ascending colon on the left side. A peritoneal fibrous band – also known as Ladd’s band – can compress duodenum causing duodenal obstruction. Intestinal malrotation is a disease of newborn as it frequently manifests in the first month of life; adult manifestation is very rare. Clinical presentation in adults is more variable [[9], [10], [11]] and can be asymptomatic, adults patients rarely present with acute midgut volvulus or internal hernias caused by Ladd’s bands. Often diagnosis of intestinal malrotation is made incidentally on routine imaging. CT scan of the abdomen has a diagnostic accuracy of 80% showing frequently superior mesenteric artery located to the right of superior mesenteric vein in malrotation [11,12]. The vague nature of these symptoms which can persist into adulthood requires a high level of clinical suspicion of intestinal malrotation [13,14]. A small number is diagnosed after an upper intestinal barium enema for investigation of gastro-oesophageal reflux and is discovered incidentally during abdominal surgery. A plain abdominal X-ray may show the characteristic “double bubble’’ but it is not reliable [13]. The absence of caecal gas shadow or the localization of small intestinal loops predominately in the right side should pose the suspicion of malrotation [15]. The standard upper gastrointestinal series may show a vertical duodenum that does not cross the midline; the entire small bowel is found in the right half of the abdomen. The accuracy of the upper gastrointestinal series is reported to be over 80% [16]. Malrotation can present itself in any form and at any age. Symptoms such as intermittent vomiting, epigastric pain and failure to thrive do not always imply a diagnosis of gastrointestinal reflux. An upper gastro-intestinal barium enema and follow through should be performed in all cases of bilious vomiting as well as in patients suffering from chronic vomiting in order to rule out malrotation.

It is still controversial if asyntomatic patients with documented malrotation require surgery. Many authors recommend elective Ladd’s procedure in all patients with intestinal malrotation [[17], [18], [19]]. Ladd’s procedure is the standard treatment for intestinal malrotation both in pediatric and adult patients. It includes division of Ladd’s band, lengthening of duodenum, widening of mesentery, derotation of midgut volvulus if present and appendectomy to prevent diagnostic dilemma due to abnormal position. Ladd’s procedure can be performed both in laparoscopy and laparotomy, but open approach is the gold standard in urgency, in suspected volvulus and bowel ischemia and gangrene [10].

## 4 Conclusion

Intestinal malrotation is a rare entity and adult presentation is even rarer: it has been reported that the incidence in adults is approximately 0.2%. Evidence from post mortem studies suggest that gut malrotation may affect up to 1 in 6000 [9,20]. Some cases are asymptomatic. In symptomatic cases two distinct patterns of adult presentations have been reported in the literature: acute and chronic [4,12,21]; chronic presentation which is more common in adults, characterised by intermittent crampy abdominal pain, bloating, nausea and vomiting over several months or years. The pathophysiology of these chronic symptoms may relate to the compression effect of Ladd’s bands running from the caecum and ascending colon to the right abdominal wall [12,14]. Acute presentation is more rare [14] and may be due to volvulus of the midgut or ileocaecum, reported as the most common cause of bowel obstruction in adults with gut malrotation. Other causes of acute presentation may be related to internal herniation caused by Ladd’s bands. Several authors have reported atypical presentations before discovering gut malrotation with abnormal location of the caecum and appendix at surgery [14,22]. We can expect an increase in the incidental diagnosis of gut malrotation with widespread use of radiological investigations. Specific findings that are diagnostic of malrotation can be detected through the use of CT scan which is considered the investigation of choice, providing diagnostic accuracy of 80% [11,12,14], both upper and lower gastrointestinal tract barium enema studies, angiography of the superior mesenteric artery, and often emergency laparotomy. Characteristic findings include the positioning of the superior mesenteric vein lying to the left or anterior to the superior mesenteric artery, the presence of a right-sided duodeno-jejunal junction, the absence of a cecal gas shadow on the patient’s right side, or a third and fourth duodenal junction that does not cross the patient’s spine [20]. On first time Fisher described a distinctive pattern in a patient with midgut volvulus, as the ‘whirlpool’ sign on CT scan due to mesentery twisted around the SMA axis [23,24].

It is argued that elective Ladd’s procedure should be performed in all patients with intestinal malrotation. The surgical treatment remains as it was first described by Ladd in 1932: mobilization of the right colon and duodenum, division of Ladd’s bands, division of adhesions around the superior mesenteric artery, appendectomy [15]. Generally, symptomatic patients with malrotation should undergo to surgical exploration. There are different management opinions: conservative or operative treatment should be discussed with the patient, highlighting all the possible risks and potential complications. Most authors are of the opinion that Ladd’s procedure is an adequate treatment for intestinal malrotation [14,22] and the elective intervention is suggested on presence of high risk anatomy.

## Conflicts of interest

None.

## Sources of funding

None.

## Ethical approval

This clinical case report is exempt from ethical approval.

## Consent

The authors obtained from the patient written informed consent for the publication of this case report and images.

## Author contribution

CG and FB were responsible for the surgical intervention. CG conceived the case report. LC and GP researched and drafted the manuscript. MDD, GMC and PC revised the manuscript. All authors read and approved the final manuscript.

## Registration of research studies

NA.

## Guarantor

Carmine Grassi.

## Provenance and peer review

Not commissioned, externally peer-reviewed.
